# Meiosis in Mice without a Synaptonemal Complex

**DOI:** 10.1371/journal.pone.0028255

**Published:** 2011-12-02

**Authors:** Anna Kouznetsova, Ricardo Benavente, Albert Pastink, Christer Höög

**Affiliations:** 1 Department of Cell and Molecular Biology, Karolinska Institutet, Stockholm, Sweden; 2 Department of Cell and Developmental Biology, Biocenter, University of Würzburg, Würzburg, Germany; 3 Department of Toxicogenetics, Leiden University Medical Center, Leiden, The Netherlands; National Cancer Institute, United States of America

## Abstract

The synaptonemal complex (SC) promotes fusion of the homologous chromosomes (synapsis) and crossover recombination events during meiosis. The SC displays an extensive structural conservation between species; however, a few organisms lack SC and execute meiotic process in a SC-independent manner. To clarify the SC function in mammals, we have generated a mutant mouse strain (*Sycp1* ^−/−^ *Sycp3* ^−/−^, here called SC-null) in which all known SC proteins have been displaced from meiotic chromosomes. While transmission electron microscopy failed to identify any remnants of the SC in SC-null spermatocytes, neither formation of the cohesion axes nor attachment of the chromosomes to the nuclear membrane was perturbed. Furthermore, the meiotic chromosomes in SC-null meiocytes achieved pre-synaptic pairing, underwent early homologous recombination events and sustained a residual crossover formation. In contrast, in SC-null meiocytes synapsis and MLH1-MLH3-dependent crossovers maturation were abolished, whereas the structural integrity of chromosomes was drastically impaired. The variable consequences that SC inactivation has on the meiotic process in different organisms, together with the absence of SC in some unrelated species, imply that the SC could have originated independently in different taxonomic groups.

## Introduction

The synaptonemal complex (SC) is a meiosis-specific protein structure found almost universally in sexually reproducing eukaryotic organisms [1], [2], [3]. Ultrastructural analysis of the SC by transmission electron microscopy has revealed a tripartite organization, with two chromosome axes (also called lateral elements – LE), surrounding a central element (CE). The axes of the two homologous chromosomes and the CE are connected along their entire length by fine fibrillar structures, the transverse filaments (TF), generating a zipper- or ladder-like structure. The TF and the CE together form the central region (CR) of the SC. The SC stabilizes presynaptic alignment of the axes of the homologous chromosomes and promotes maturation of crossover recombination events, generating physical linkages between bivalents (chiasmata). A failure to establish chiasmata gives rise to achiasmatic chromosomes (univalents), which increases the risk of chromosome missegregation at the first meiotic cell division, and formation of aneuploid germ cells [4].

SYCP1 represents a major constituent of the TF and is essential both for recruiting CE proteins to the SC and for synapsis [5]. Besides SYCP1, four proteins have been shown to contribute to the formation of the CE of the SC in mouse meiocytes: SYCE1, SYCE2, SYCE3 and TEX12. Inactivation of these genes allows for SYCP1 loading, but impede formation of the continuous CR structure connecting homologous chromosomes and maturation of crossover recombination intermediates [6], [7], [8], [9]. The LE proteins SYCP2 and SYCP3 in mice contribute to the organization of the meiotic chromosome axis together with the cohesin complex proteins that mediate sister chromatid cohesion [10], [11] and the HORMA domain proteins that promote early recombination events and synapsis [12], [13], [14]. Importantly, the cohesin complex proteins and the HORMA domain proteins remain associated with the meiotic chromosome axis in the absence of SYCP2 and SYCP3 (and the LE), which shows that the axis is composed of several independent organizational layers [13], [15].

A comparison of the SC between species gives an enigmatic picture: at the ultrastructural level the formation is highly conserved, but significant differences appear at the molecular level. This variability between species involves both the number of identified SC proteins and the conservation of the primary sequences of functionally related proteins. To date, seven SC proteins have been identified in mouse *Mus musculus*, four in worm *Caenorhabditis elegans*, three in fly *Drosophila melanogaster* and two in yeast *Saccharomyces cerevisiae*, cress *Arabidopsis thaliana* and rice *Oryza sativa*. Assigning the known SC proteins to the three different structural entities of the SC: the LE, the TF and the CE, is not trivial. Five different LE proteins are known: SYCP2 and SYCP3 in mice [16], [17], Red1 in *S. cerevisiae* [18], ORD in *D. melanogaster* [19]) and PAIR3 in *O. sativa* [20]. Ten TF proteins have been identified: SYCP1 [5] in mice, Zip1 in *S. cerevisiae* [21], C(3)G in *D. melanogaster* [22], SYP-1, SYP-2, SYP-3 and SYP-4 in *C. elegans* [23], ZYP1a and ZYP1b in *A. thaliana* [24], [25] and ZEP1 in *O. sativa* [20]. Furthermore, five different CE proteins have been recognized: SYCE1, SYCE2, SYCE3 and TEX12 in mice [9], [26], [27], and CONA in *D. melanogaster* [28]. Studies of mutants in yeast, *C. elegans* and *D. melanogaster*, in which SC formation has been abolished, have provided important information about the function of the SC. Here we describe for the first time a mammalian model system in which SC formation has been abolished. We have generated a mouse strain in which all known SC proteins (i.e. SYCP1, SYCP2, SYPC3, SYCE1, SYCE2, SYCE3 and TEX12) have been simultaneously displaced from the meiotic chromosomes. We have studied structural as well as molecular aspects of the meiotic process in spermatocytes and oocytes in this mouse strain, to further understand the functions of the SC.

## Results

### Synaptonemal complexes are not formed in *Sycp1^−/−^Sycp3^−/−^* double-null germ cells

Inactivation of the gene encoding SYCP3 disrupts the loading of SYCP2 onto the meiotic chromosome axis [17]. Similarly, inactivating the gene encoding SYCP1 abolishes the recruitment of SYCE1, SYCE2, SYCE3 and TEX12 to the central region of the SC [6], [7], [8], [9]. We took advantage of this and generated *Sycp1^−/−^Sycp3^−/−^* double-null mice to study meiotic progression in SC-deficient germ cells.

**Figure 1:**
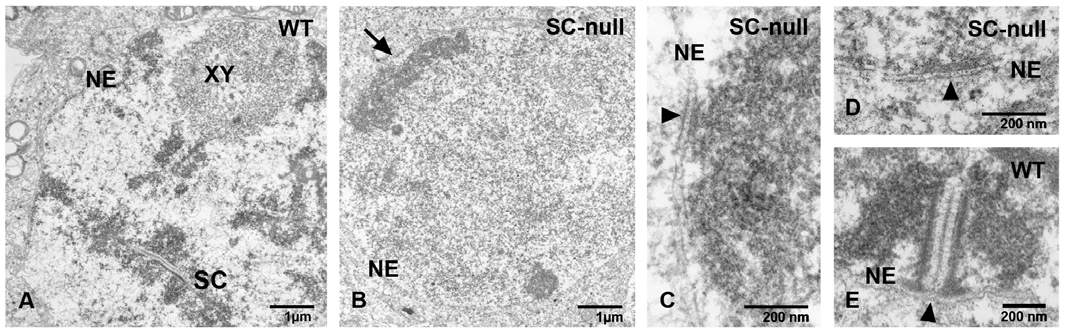
SC-null spermatocytes lack nuclear structures resembling the axial elements or the central region of the SC. Electron microscopy analysis of nuclei from spermatocytes derived from of wild-type (A, E) and SC-null (B–D) mice. Wild-type pachytene meiocytes show synaptonemal complexes (SC) and normally condensed chromatin (A, E). In SC-null meiocytes, chromatin is less condensed and axial structures are absent (B). The arrow in (B) points to dense regions of centromeric heterochromatin located close to the nuclear envelope. Attachment plates of the nuclear envelope in wild-type and SC-null meiocytes are denoted by arrowheads (C–D). NE, nuclear envelope; XY, XY body.

We initially analyzed *Sycp1^−/−^Sycp3^−/−^* double-null spermatocytes by transmission electron microscopy. Neither SCs nor its individual structural entities (LEs, TFs or CEs) were observed in the mutant cells (Fig. 1). We therefore would refer to *Sycp1^−/−^Sycp3^−/−^* double-null meiocytes as SC-null later on. No chromosome axis corresponding to the cohesin cores was seen in SC-null spermatocytes and chromatin in the mutant cells appeared less condensed and more homogeneously distributed (Fig. 1A,B). In wild-type meiocytes, the distal ends of the SC, including the LEs and the CR, were firmly connected to attachment plates situated at the nuclear envelope (Fig. 1E, [29]). In the absence of the SC, we found that seemingly unorganized chromatin fibers remained in contact with the attachment plates (arrowheads in Fig. 1C,D). In summary, we find that the SC, including the LE, the TF and the CE, are not formed in SC-null spermatocytes. The residual chromosome organization that exists in SC-null cells is, however, sufficient to maintain a connection between the telomere regions of the chromosomes and the attachment plates at the nuclear envelope. This suggests that telomeric DNA sequences establish a direct contact with the attachment plates and that the SC acts as a non-essential supporting framework.

### The SC is required for both synapsis and the structural integrity of the chromosome axis

**Figure 2:**
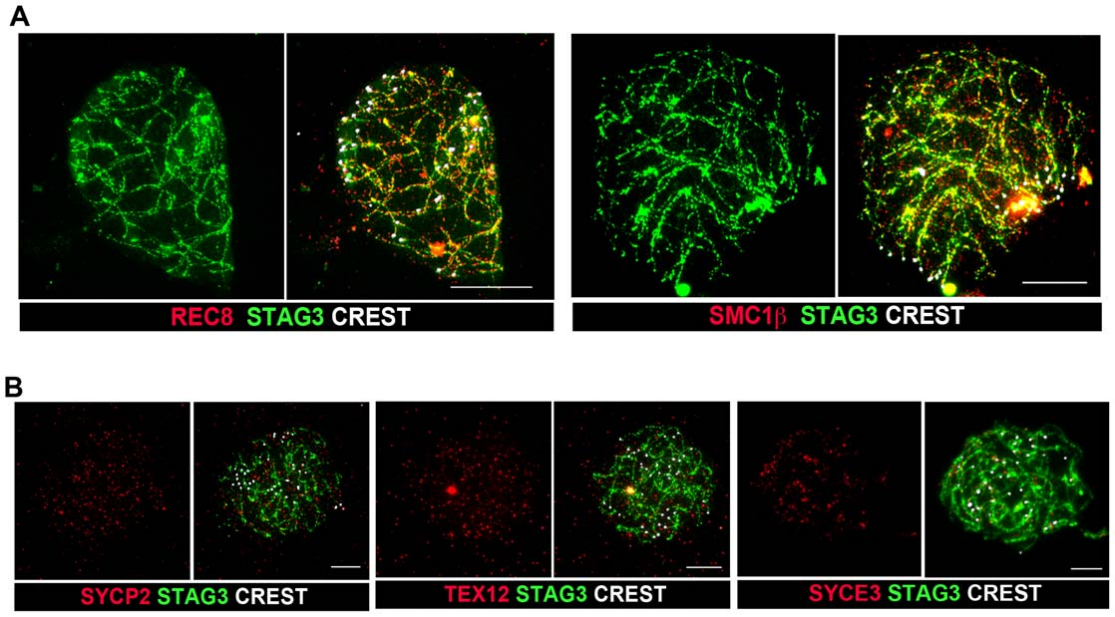
SC proteins, but not cohesin proteins, are lost from the chromosome axes in SC-null spermatocytes. (A) SC-null spermatocytes were stained with antisera against the meiosis-specific cohesins REC8 (red) and SMC1β (red) and the cohesin protein STAG3 (green). Centromeres, labeled by CREST, are shown in white. (B) SC-null spermatocytes were labeled with antisera against the axial element protein SYCP2 (red), the central element protein TEX12 (red) or the central element protein SYCE3 (red). The chromosomal axes are identified by labeling of the cohesion protein STAG3 (green). Centromeres, labeled by CREST, are shown in white. Bars, 10 µm.

To define how the loss of the SC impacts on other chromosome-associated protein complexes, we used immunostaining. In SC-null spermatocytes, labeled antibodies against SYCP2, SYCE3 and TEX12 did not reveal chromosome-axis associated structures (Fig. 2B). But, antibody staining for STAG3, REC8 and SMC1β displayed residual axial chromosome structures in SC-null spermatocytes (Fig. 2A), which also retained HORMAD1-staining (T. Fukuda, pers. communication). Further analysis of meiotic progression in SC-null spermatocytes, however, was prohibited as male germ cells are eliminated at spermatogenic stage IV (the zygotene/early pachytene stage of prophase I) [7].

**Figure 3:**
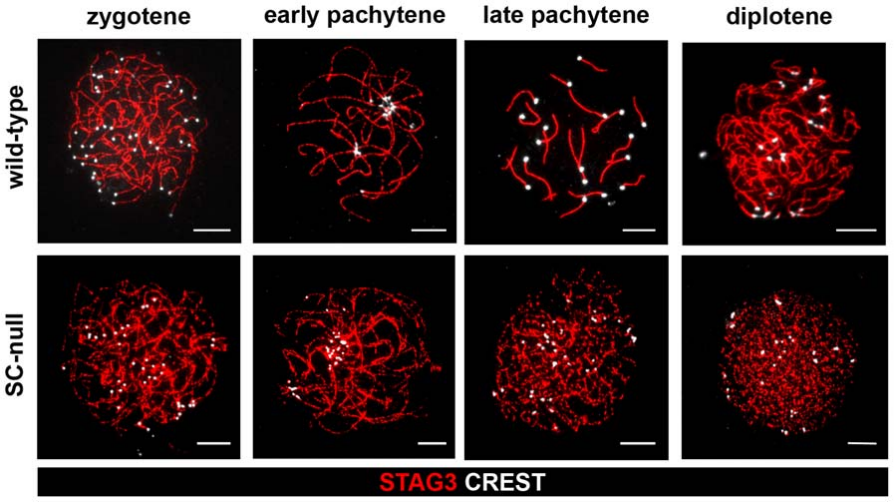
An axial cohesin structure develops in the absence of the SC. SC-null and wild-type oocytes from prenatal ovaries were labeled with antisera that recognize the cohesion complex (STAG3-red) and the centromeres (CREST-white), and analyzed by immunofluorescent microscopy. Mutant oocytes were classified according to their day of appearance during development (see Materials and Methods). Cohesin cores that showed extensive alignment were found in SC-null oocytes. The cores failed to synapse, as judged by the number of CREST foci seen at late pachytene. The integrity of the cohesion axes rapidly declined from the pachytene stage and onwards in SC-null oocytes. Bars, 10 µm.

**Figure 4:**
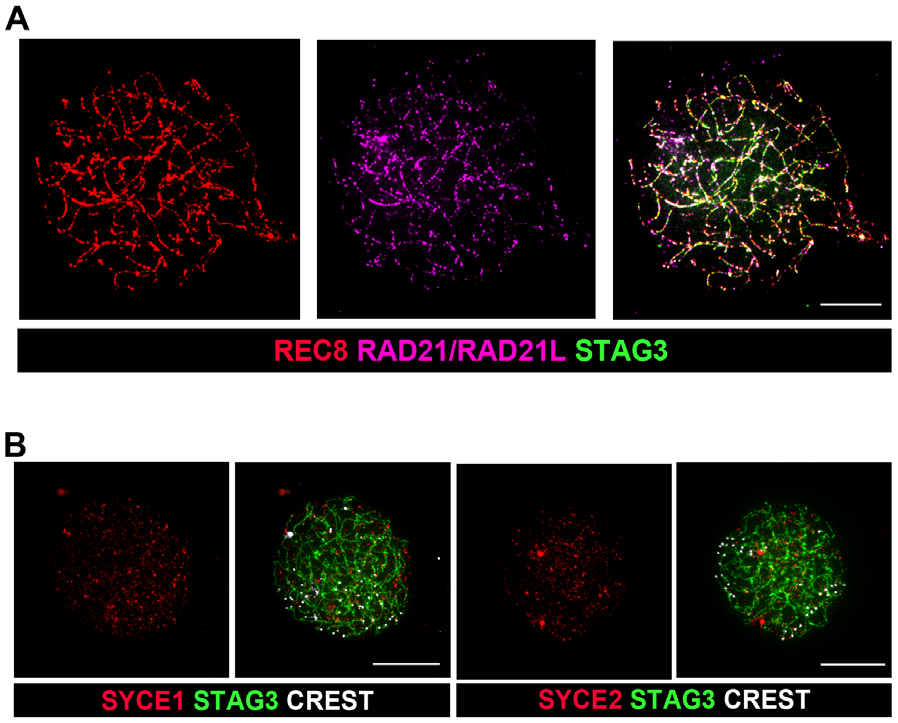
Cohesin proteins label the chromosomal axes in SC-null oocytes, but the SC proteins are lost from the chromosomal axes. (A) SC-null oocytes were stained with antisera recognizing cohesins REC8 (red), RAD21/RAD21L (magenta) and STAG3 (green). (B) SC-null oocytes were labeled with antisera against the central element proteins SYCE1 and SYCE2 (red). Chromosomal axes were identified by labeling of the cohesion protein STAG3 (green). Centromeres, labeled by CREST, are shown in white. Bars, 10 µm.

Instead, we analyzed the meiotic process in SC-null oocytes and found that their progression through meiosis was not blocked (Fig. 3; for staging of meiosis in SC-null oocytes, see Materials and Methods). Immunostaining of SC-null oocytes at the zygotene stage of prophase I by antibodies against cohesin complex proteins REC8, RAD21/RAD21L, STAG3, SMC1β and SMC3 identified axial chromosome cores, similar to those observed in spermatocytes (Fig. 4A and data not shown). Again, antibodies against the SC proteins SYCP2, TEX12, SYCE1 and SYCE2 did not label these chromosome cores (Fig. 4B and data not shown). The residual chromosome axes were found to display presynaptic pairing at the zygotene stage in the mutant oocytes and we detected 40 individual centromeres in the SC-null oocytes (Fig. 3). The clustering of the centromeres suggests that the bouquet formation process is intact in the absence of SC. No evidence for synapsis of the axial cohesin cores was found in SC-null oocytes. Progression through the pachytene and diplotene stages in SC-null oocytes resulted in extensive fragmentation of the axial cohesin cores (Fig. 3), similar to what is seen in SYCP3-null oocytes [30], strongly suggesting that their integrity depend on formation of the LEs. Our results show that the SC is not required for pairing of the centromeres of the sister chromatids, bouquet formation, recruitment of cohesin-complex or HORMAD-domain proteins to the chromosome axis or for presynaptic alignment of the axial cohesin cores. We found instead that the SC is essential for synapsis and the preservation of the structural integrity of the chromosome axes.

### Repair of meiotic DNA double-strand breaks is impaired in SC-null oocytes and MLH1-MLH3-dependent crossovers are not generated

**Figure 5:**
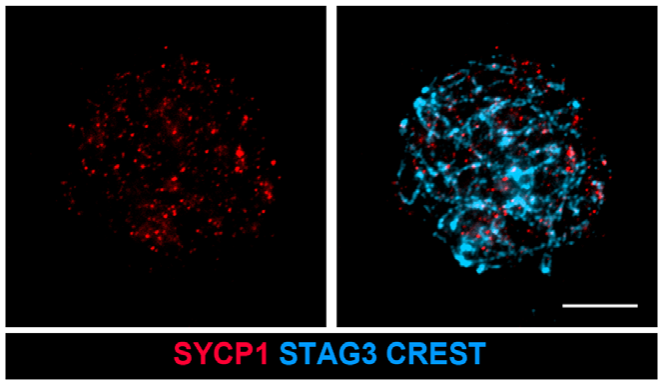
The SYCP1 protein does not form extended fiber-like structures in TEX12/SYCP3 double-null oocytes. Mutant oocytes were labeled with antisera against SYCP1 (red) and STAG3 (blue). Centromeres were identified by CREST staining (blue). Bar, 10 µm.

Meiotic recombination occurs in the context of the synaptonemal complex [2]. We therefore investigated by immunofluorescence microscopy the recombination process in SC-null oocytes, using a set of temporally overlapping markers. The results were compared to those for wild-type oocytes and SYCP1-null oocytes. We also analyzed TEX12-null and TEX12/SYCP3 double-null mutant oocytes to define the recombination defects that depend on the integrity of the SC *per se*, rather than the absence of SYCP1 protein. The meiotic chromosomes in SYCP1-null oocytes do not synapse, while chromosomes in TEX12-null oocytes show partial synapsis with short TF regions distributed along the otherwise asynapsed homologous chromosomes [7]. The TEX12/SYCP3 double-null oocytes assemble cohesin cores and express SYCP1, but do not synapse, show organized TFs or assemble CE structures (Fig. 5).

**Figure 6:**
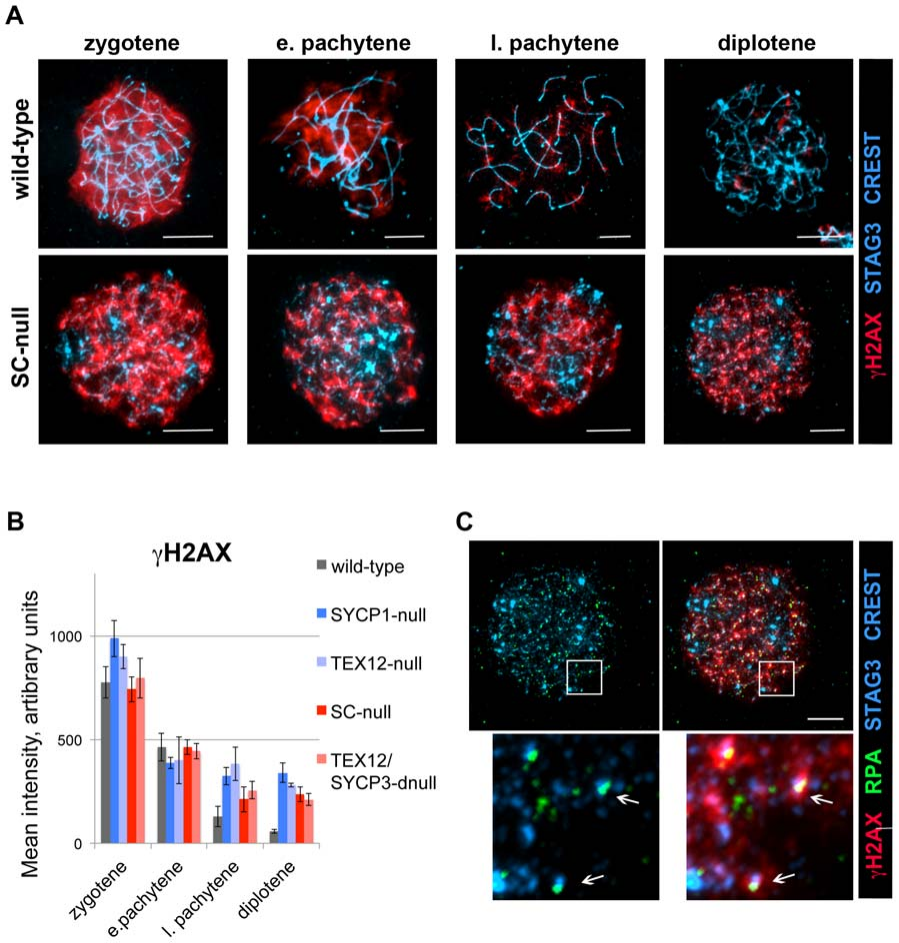
Repair of DNA DSBs are impaired in the absence of the SC. SC-null and wild-type oocytes from prenatal ovaries were labeled with antisera that recognize the cohesion complex (STAG3-blue) and the centromeres (CREST-blue), and analyzed by immunofluorescent microscopy. (A) H2AX phosphorylation (red) persists in SC-null oocytes until the diplotene stage. (B) Mean intensity of the γH2AX signal in nuclei of wild-type and mutant oocytes. (C) RPA foci (green) co-localize with γH2AX (red) in diplotene stage SC-null oocytes. Bars, 10 µm.

Meiotic recombination is initiated by the introduction of double strand breaks (DSBs) into DNA [4]. Formation of the DSBs along the chromosome axis during meiosis can be monitored by the temporal appearance of a phosphorylated form of H2AX (γH2AX) [31]. γH2AXfoci formation was observed at similar levels in wild-type and SC-null zygotene oocytes (Fig. 6A,B). Most of the γH2AXsignal was lost at the diplotene stage in wild-type oocytes, whereas a strong residual γH2AXsignal remained in SC-null oocytes at late meiotic stages (Fig. 6A,B). A similar level of residual γH2AX staining was also observed in SYCP1-null, TEX12-null and TEX12/SYCP3 double-null oocytes, strongly suggesting that the repair process depends on an intact CE of the SC.

**Figure 7:**
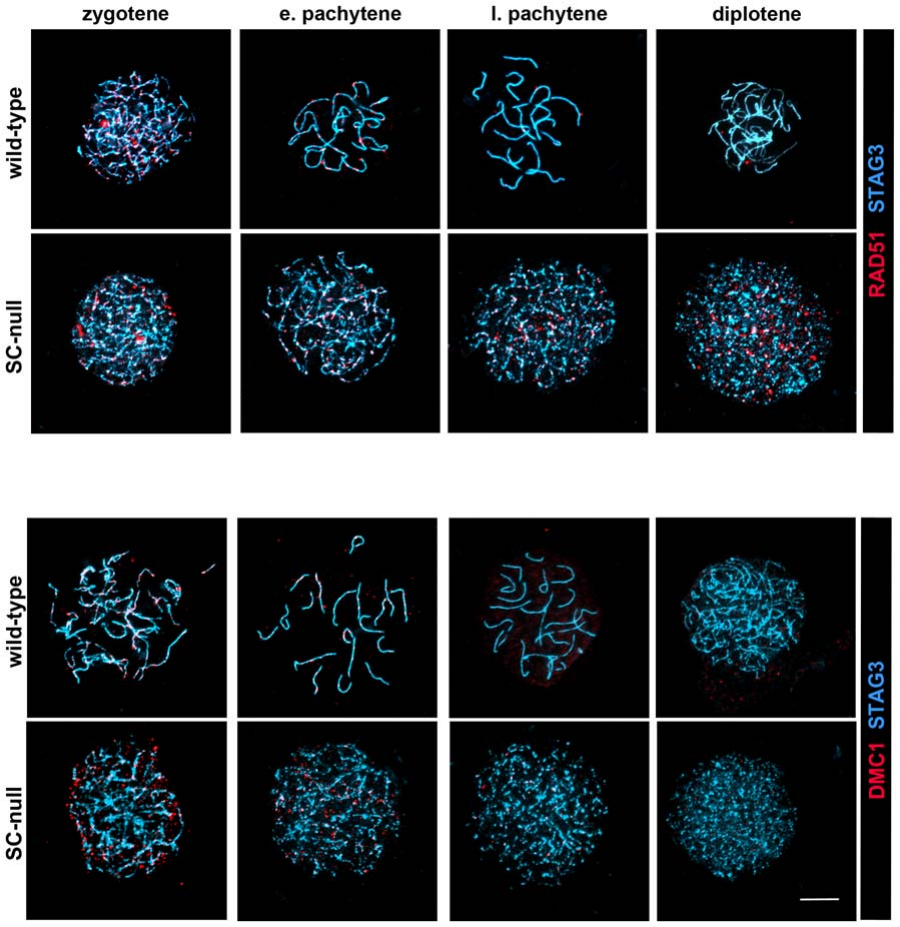
Temporal expression of RAD51 and DMC1 in wild-type and SC-null oocytes. Oocytes at different stages of meiotic prophase were labeled with antisera to RAD51 or DMC1 (red). The chromosomal axes were visualized by STAG3 protein labeling (blue). The RAD51 and DMC1 recombination-related proteins disappear from the chromosomal axes in wild-type oocytes by late pachytene. In SC-null oocytes DMC1 show a pattern similar to that seen in wild-type oocytes, while RAD51 foci persist until the diplotene stage. Bar, 10 µm.

To provide more insight into the cause of this repair defect, we monitored the temporal appearance and disappearance of DNA repair proteins that take part in the conversion of DNA DSBs into crossovers, including Replication Protein A (RPA), DNA repair protein RAD51, meiotic recombination protein DMC1, MutS protein homolog 4 (MSH4) and DNA mismatch repair proteins MLH1 and MLH3 [4]. We found that the chronological appearance of foci representing RAD51, DMC1, RPA and MSH4 on chromosomes during meiosis was the same in wild-type and in the four different mutant oocytes (Figs. 7–9). This suggests that DNA DSBs formation, as well initiation of DNA DSBs repair processes such as DNA strand exchange (promoted by RAD51, DMC1 and RPA [32]) and generation of Holliday junctions (recognized by MSH4 [33]), is functionally intact in the mutant oocytes, despite the absence of a SC.

**Figure 8 pone-0028255-g008:**
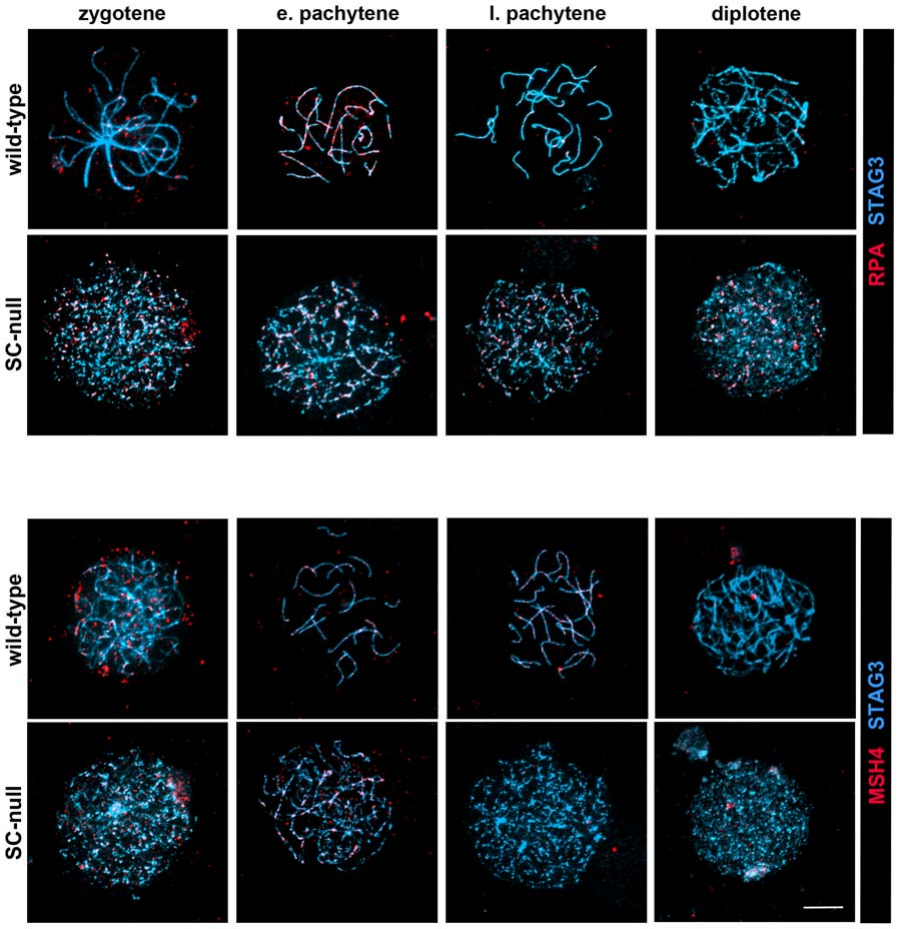
Temporal expression of RPA and MSH4 in wild-type and SC-null oocytes. Oocytes at different stages of meiotic prophase were labeled with antisera to RPA or MSH4 (red). The chromosomal axes were visualized by STAG3 protein labeling (blue). The RPA and MSH4 recombination-related proteins disappear from the chromosomal axes in wild-type oocytes by late pachytene. In SC-null oocytes MSH4 shows a pattern similar to that seen in wild-type oocytes, while RPA foci persist until the diplotene stage. Bar, 10 µm.

**Figure 9:**
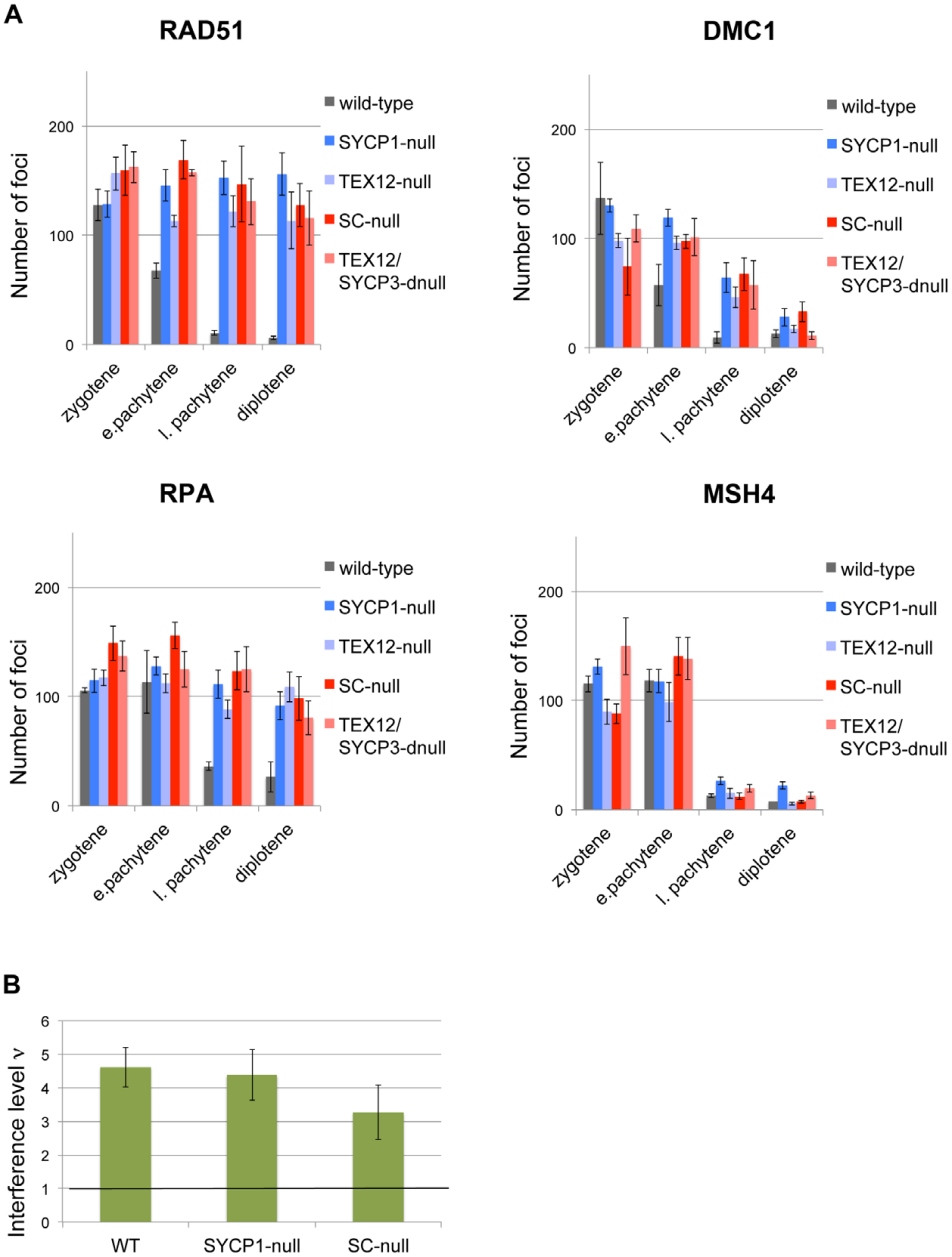
The DNA recombination process is correctly initiated in SC-deficient oocytes, but the repair process is severely obstructed. The temporal and spatial distribution of RAD51, DMC1, RPA and MSH4 was analyzed at different stages of meiosis in wild-type, SYCP1-null, TEX12-null, TEX12/SYCP3 double-null and SC-null ovaries. (A) The number of axis-associated RAD51, DMC1, RPA and MSH4 foci in wild-type and mutant oocytes was revealed using immunofluorescent microscopy (Figs. 7–8) and scored. The recombination-related proteins disappear from the chromosomal axes in wild-type oocytes by late pachytene. In mutant oocytes, DMC1 and MSH4 show a similar turnover, while RAD51 and RPA persist to the diplotene stage. (B) The level of interference between MSH4 foci is similar in wild-type, SYCP1-null and SC-null oocytes, as estimated by the value of shape parameter νof gamma-distribution. A value of 1 indicates the absence of interference. Bars, s.e.m.

In agreement with the delayed removal of γH2AX from chromosomes in mutant oocytes, RAD51 and RPA were found to remain on the axis of the chromosomes in SC-null oocytes even at late meiotic stages (Figs. 7–9). Furthermore, RPA was found to co-localize with γH2AX at the pachytene and diplotene stages in SC-null oocytes (Fig. 6C), supporting the presence of unrepaired DSBs in these cells. Surprisingly, DMC1 and MSH4 were found to be lost from chromosomes in SC-mutant oocytes in a temporal pattern similar to the one observed in wild-type oocytes (Figs. 7–9).

**Figure 10:**
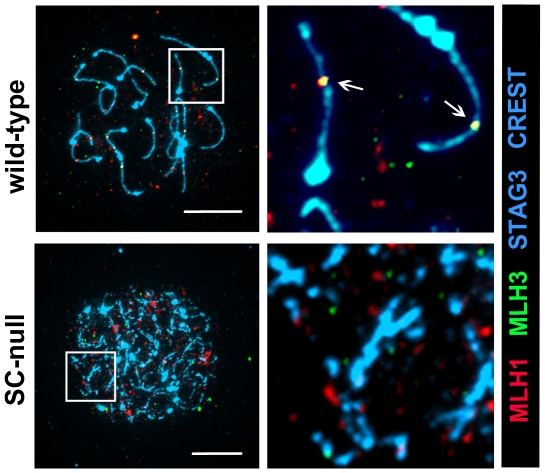
The recombination process in SC-null oocytes stops prior to the formation of recombination structures that contain MLH1 or MLH3. The chromosomal axes were labeled by STAG3 (blue), and centromeres by CREST (blue). MLH1 (red)/MLH3 (green) axis-associated complexes are absent in SC-null oocytes at the pachytene stage. Bars, 10 µm.

We next labeled SC-null oocytes with antibodies against the late recombinant markers, MLH1 and MLH3 (MutL homologs 1 and 3, respectively), which co-localize at the sites of class I crossovers [34]. However, no overlapping MLH1 and MLH3 foci could be observed on the chromosomal cores of SC-null oocytes (n = 153) (Fig. 10). Thus, the impaired synapsis and repair processes in SC-null oocytes block formation of a class I crossovers, as visualized by the absence of MLH1 and MLH3 foci.

Class I crossovers display positive interference, i.e. the occurrence of a crossover inhibits formation of additional crossovers in adjacent chromosomal regions. Positive interference is retained for the crossover precursors at the zygotene stage in the absence of TFs in SYCP1-null meiocytes [35], as well as in the absence of LEs in SYCP3-null oocytes [36]. We analyzed whether positive interference was also retained in SC-null oocytes. The strength of interference was measured by fitting the frequency distribution of the interfocal distances for MSH4 foci to the gamma distribution [35]. The shape parameter of the gamma model (ν) measures the strength of interference. We found that the interference level for MSH4 foci in SC-null oocytes did not differ significantly from that found in wild-type and SYCP1-null oocytes (Fig. 9B). The wild-type level of positive interference between MSH4 foci observed in SC-null oocytes supports the idea that the sites of future crossovers are pre-defined early in prophase before SC formation [37]. However, since only a subset of the MSH4 foci is converted into MLH1-containing mature recombination nodules, it remains possible that the SC is essential for establishing additional levels of crossover interference imposed at a later stage.

### Oocyte loss is transiently suppressed during early postnatal development in SC-null oocytes compared to SYCP1-null oocytes

**Figure 11:**
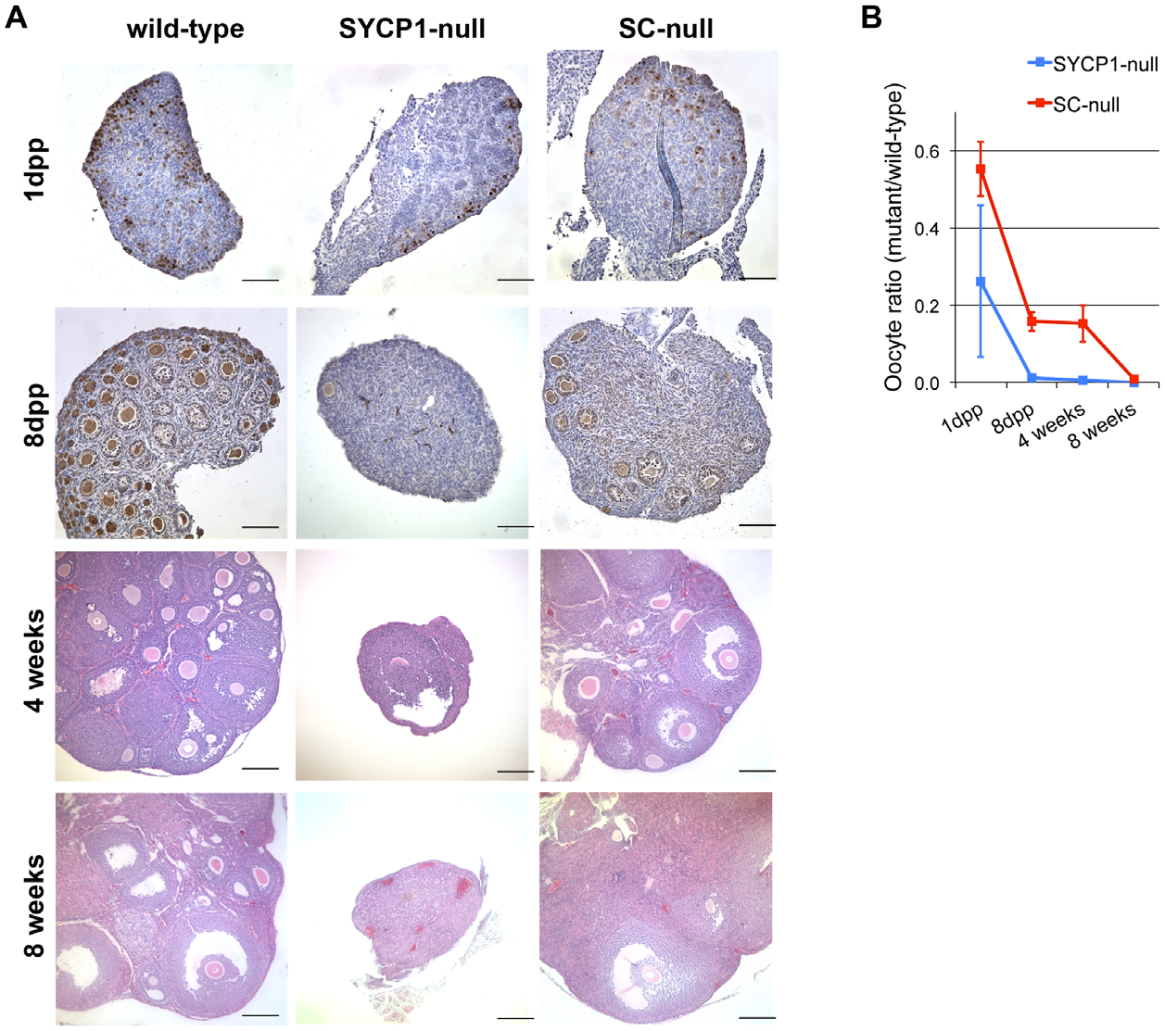
Inactivation of the SYCP3 gene in a SYCP1-null background transiently suppresses oocyte loss. (A) Sections of ovaries taken from mutant and wild-type females were stained by GCNA (at 1dpp and 8dpp), or with hematoxylin and eosin (at 4 weeks and 8 weeks). Bars, 100 µm. (B) Oocyte numbers in wild-type and mutants animals and the ratio of mutant/wild-type oocytes were scored at 1 day (1 dpp), 8 days (8 dpp), 4 weeks and 8 weeks after birth. Bars, s.d.

The impaired DNA repair process identified in SC-null oocytes results in recombination intermediates that remain at the diplotene stage of prophase I, aberrant structures that could impact on oocyte viability and thus cause problems with fertility. To address this issue, SC-null female mice were mated with wild-type males; however, no pups were generated (data not shown). To find out the cause of infertility, we examined ovary morphology in SC-null animals. No differences in ovary size or oocyte numbers were found in wild-type, SYCP1-null or SC-null females at day 16.5 of embryonic development (data not shown). We next compared oocyte numbers in ovaries derived from the two different null genotypes at different time points of early postnatal development. We found that the ovaries of SC-null females at the first day post partum (1 dpp), at 8 dpp and at 28 dpp were larger than the ovaries seen in the SYCP1-null animals at the same time point, and contained significantly more oocytes (Fig. 11A,B). However, all oocytes were subsequently lost in 8-week old SC-null animals. Thus, elimination of SYCP3 in a SYCP1-null background transiently suppresses oocyte loss during ovarian development.

### Chiasmata are formed in SC-null oocytes in the absence of synapsis and MLH1-dependent crossovers

**Figure 12:**
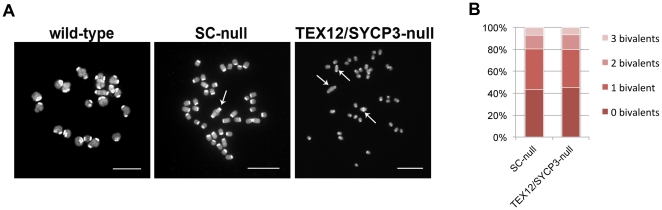
A small number of chiasmata forms in SC-null oocytes. (A) Wild-type, SC-null and TEX12/SYCP3 double-null oocytes at the first meiotic metaphase stage were stained with DAPI. Arrows indicate bivalents. The occurrence of bivalents strongly suggests that homologous chromosomes are held together by chiasmata. Bars, 10 µm. (B) Percentage of SC-null (n = 55) and TEX12/SYCP3 double-null (n = 57) oocytes that contain 0–3 bivalents per cell.

We next analyzed if the immature oocytes in 4-week-old SC-null animals could be induced to mature to the meiosis I (MI) stage. Oocytes isolated from the ovaries of 4-week-old wild-type and SC-null females were cultured in vitro for six hours. We found that the absence of the SC does not block oocyte maturation and oocytes derived from SC-null ovaries could develop to the MI stage. Chromosome analysis revealed no difference between wild-type and SC-null chromosomes at the MI stage when compaction levels or centromere structures were compared (Fig. 12A), but a large majority of the chromosomes in SC-null oocytes appeared as univalents, in contrast to the situation in wild-type oocytes. Interestingly, more than 50% of the SC-null oocytes contained one to three bivalents that were held together by chiasmata, despite a lack of synapsis and the absence of detectable MLH1/MLH3 foci at the preceding pachytene stage in the mutant oocytes (Fig. 12A,B). The number of bivalents was not increased by the residual presence of SYCP1, as a similar number of bivalents were observed also in TEX12/SYCP3 double-null oocytes (Fig. 12A,B).

## Discussion

We have generated and characterized the first SC-null mutant in mammals by generating *Sycp1* ^−/−^ *Sycp3* ^−/−^ double knockout mice. The results of this analysis are summarized in Table 1, together with the previously known data for the *Sycp1* ^−/−^ and *Sycp3* ^−/−^ single mutants. The SC structure was found to be dispensable for attachment of telomeres to the nuclear envelope, for recruitment of cohesin complex proteins and HORMAD-domain proteins to the chromosome axes, for pairing of sister centromeres, for formation of DNA DSBs, for loading of recombination proteins such as RAD51, DMC1, RPA and MSH4 onto chromosomes and for establishing positive interference. In contrast, the SC is essential for synapsis, for maintenance of chromosome pairing, for repair of the recombination intermediates, for stabilization of Holliday junctions between homologous chromosomes and for generation of MLH1-dependent crossovers. Notably, the chromosomal cores formed by cohesin proteins in SC-null oocytes rapidly disintegrate during meiosis, similar to what is seen in SYCP3-null oocytes [30], strongly suggesting that their integrity depend on formation of the LEs. Mutants of the *D. melanogaster* LE protein, ORD, also demonstrate premature disassembly of the cohesion cores [38].

**Table 1:** Phenotypes identified for SYCP1-null, SYCP3-null and SC-null mutant mice.

	SYCP1-null	SYCP3-null	SC-null
SC formation	no CR	no LE, aberrant CR	no LE, no CR
Nuclear envelope attachment	yes	yes	yes
Synapsis	no	partial	no
Cohesin/HORMAD-domain proteins core formation	yes	yes	yes
Positive interference	yes^1^	yes^2^	yes^3^
Repair of recombination intermediates	delayed	delayed	delayed
MLH1-dependent crossovers formation	no	yes	no
Number of oocytes at 8dpp(% of the wild type)	0%	30%	15%
Fertility	no	partial	no

1 shown for MSH4 protein foci.

2 shown for MLH1 protein foci.

3 shown for MSH4 protein foci.

**Figure 13:**
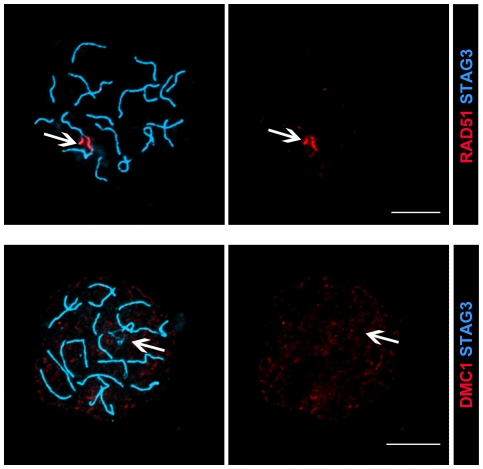
RAD51 (red), but not DMC1 (red), is found on the asynapsed axes in wild-type oocytes. Chromosomal axes are labeled by STAG3 (blue). Bars, 10 µm.

We found that DMC1 and MSH4, in contrast to RAD51 and RPA, were displaced from asynapsed chromosomes of SYCP1-null and SC-null oocytes in a pattern similar to what was seen for these proteins in wild-type oocytes. This suggests that the continued DNA strand exchange activity of DMC1 and the retention of MSH4 at Holliday junctions require an aspect of SC function, involving either a stable close alignment of homologs or a direct physical association with CR components of the SC. The first possibility seems more likely for several reasons. In yeast, Dmc1 is essential for creating inter-homologue recombinants, while Rad51 is required for inter-sister recombination [39]. Thus synaptic failure, as seen in SC-null and SYCP1-null oocytes, most likely blocks further DMC1 action and results in the displacement of this protein from chromosomes. The dependence of close homolog alignment for the maintenance of DMC1 on chromosomes is also supported by its absence from asynapsed chromosomes in wild-type oocytes (Fig. 13). In contrast, sister chromatid pairing is intact in SC-null oocytes, and therefore does not affect RAD51 binding to the asynapsed chromosomes in these cells. In vitro studies of MSH4 have shown that it binds specifically to the core of Holliday junctions [33]. The absence of synapsis in SC-null oocytes most likely generates considerable stress on the Holiday junctions established between homologous chromosomes at the zygotene stage, resulting in a premature loss of MSH4 from the chromosomes.

Unexpectedly, we found that 57% (n = 55, s. d. = 14%) of SC-null and 55% (n = 57, s. d. = 6,5%) of the TEX12/SYCP3 double-null oocytes contained 1–3 chiasmata per oocyte. This corresponds to the number of chiasmata observed in MLH1- and MLH3-null oocytes [40], [41]. The chiasmata found in MLH1- and MLH3-null oocytes are probably formed by alternative MLH1-independent pathway(s), responsible for the formation of non-interfering class II crossovers, and generating 5–10% of the total crossover numbers in mice [42]. The MLH1-independent crossover-generating pathway, therefore, does not depend on the presence of a SC, in contrast to the MLH1-MLH3 pathway.

A comparison between SYCP1-null and SC-null oocytes showed a higher survival rate during prenatal development (1 dpp) and also early postnatal development (4 weeks). This suggests that loss of SYCP3 function (and the integrity of LEs of the SC) weakens the efficiency of the quality control mechanisms operative in oocytes. The LEs of the SC may directly perform surveillance functions, so their absence in SC-null oocytes prevents elimination of cells with DNA lesions. Alternatively, the LEs of the SC might provide a barrier against DSBs repair using sister chromatids as a template [43], [44], as also suggested by analysis of the *D. melanogaster* ORD mutant [19]. The consequence of this is that, on elimination of the LE in a SYCP1-null background, recombination intermediates that would otherwise remain unrepaired, could be repaired using sister chromatids as templates. This would also explain why a large fraction of the SYCP3-null oocytes are viable and contribute to the fertilization process [45].

The generation of a SC-null mouse mutant provides us with an opportunity to evaluate the function of this highly conserved protein structure in different taxonomic groups, including mammals. We have compared a set of features linked to SC function in four organisms in which formation of the SC has been experimentally abolished, including *S. cerevisiae* [46], [47], *C. elegans* [48], [49], [50], *D. melanogaster* [51], [52] and mice (this study) (Table 2). The comparison reveals that pre-synaptic pairing and axial cohesion core formation were not affected by the absence of the SC in these organisms. Importantly, however, for the other phenotypes analyzed, there was a considerable difference resulting from SC loss between organisms. Chiasmata formation was abrogated in the absence of the SC in three of the organisms, but not in *S. cerevisiae*. DSBs formation was not affected in *C. elegans* and the mouse, whereas the level of DSBs formation was considerably reduced in *S. cerevisiae* and *D. melanogaster*. Furthermore, whereas meiotic progression to the MI stage was not affected *S. cerevisiae* and *C. elegans*, this process was severely impaired in the mouse. In summary, the comparisons in Table 2 show that abrogated SC formation result in a highly variable set of phenotypes, many of them not shared between different organisms.

**Table 2:** Phenotypes described for SC-null mutants.

	*S. cerevisiae*	*C. elegans*	*D. mel* (female)	*M. musculus*
Gene(s) mutated	*Red1*	*SYP-2* 1	*C(3)G*	*Sycp1;Sycp3*
SC formation detected by EM	no	no	no	no
Pre-synaptic pairing	present	present	n/c^2^	present
DSBs formation (% of wild type level)	25%	normal	21%^3^	normal
DSBs repair kinetics	normal	delayed	normal	delayed
Chiasma formation (% of wild type level)	25%	0%	2%^4^	<10%
Progression to the MI stage(% of wild type level)	100%	93%	n/c^2^	15%
Axial cohesin core formation	yes, shown for Rec8	yes, shown for REC-8	yes, shown for C(2)M	yes, shown for STAG3, REC8, SMC1β, SMC3, RAD21/RAD21L

1 SYP-1-null and SYP-1/SYP-2 double-null mutants showed similar phenotypes to SYP-2-null.

2 not characterized.

3 in a DSB-deficient background.

4 on chromosome 3.

The similarity of the meiotic process in different eukaryotic organisms suggests that meiosis arose once early in the evolution of eukaryotes [53], [54]. The highly conserved ultrastructural organization of the SC found in organisms that belong to different taxonomic groups implies that this structure also have a single evolutionary origin. However, the absence of sequence similarity for the SC proteins between different taxonomic groups, the striking variability in subunit composition for the SC, as well as the pleiotropic consequences on meiosis seen in different organisms after SC inactivation, raise the question if indeed SC arose only once in evolution. Furthermore, the existence of entirely SC-independent meiotic processes in unrelated organisms like yeast *Schizosaccharomyces pombe* [55], the ciliate protist *Tetrahymena thermophila* [56] or the fungi *Aspergillus nidulans* [1], further challenges the concept of a single evolutionary origin for the SC.

## Materials and Methods

### Ethics statement

All animal experiments were approved by the Stockholm-North Animal Ethical Committee (application number 181/09).

### Mice

The derivation of *Sycp1^−/−^*, *Sycp3^−/−^* and *Tex12^−/−^* mouse lines has been described previously [5], [17], [27]. Spermatocytes were isolated from adult male testes. To obtain oocytes at meiotic prophase stage, heterozygote animals were mated and the females were then examined for vaginal plugs (day 0.5 of embryonic development, E0.5). Oocytes were isolated from embryos at E16.5–E19.5.

### Transmission electron microscopy

Testes of wild-type and *Sycp1* ^−/−^ *Sycp3* ^−/−^ mice were fixed in 2.5% glutaraldehyde (1 h, 4°C) and then postfixed with 1% osmium tetroxide (1 h), as described previously [9]. After overnight staining with 0.5% uranyl acetate, testes were dehydrated in an ethanol series and embedded in Epon. Ultrathin sections were double stained with uranyl acetate and lead citrate according to the standard procedures [9]. Micrographs were obtained with an EM-10 electron microscope (Carl Zeiss, Jena, Germany).

### Immunofluorescence microscopy

Slides with oocytes or spermatocytes from wild-type, *Sycp1* ^−/−^, *Tex12* ^−/−^, *Tex12* ^−/−^ *Sycp3* ^−/−^ and *Sycp1* ^−/−^ *Sycp3* ^−/−^ animals were fixed in 1% PFA using a “dry-down” technique [57] and immunostained as described previously [30]. For protein detection and quantification, we used guinea pig anti-STAG3 [30] at 1∶100, human anti-CREST at 1∶1000, rabbit anti-γH2AX (Upstate Biotechnology) at 1∶100, mouse anti-RAD51 (AnaSpec) at 1∶50, mouse anti-DMC1 (Abcam) at 1∶50, rabbit anti-RPA (gift from P. Moens) at 1∶500, mouse anti-MLH1 (Oncogene) at 1∶100, rabbit anti-MHS4 (Abcam) at 1∶50, rabbit anti-MLH3 (gift from P. Cohen) at 1∶50. Secondary antibodies were swine-anti-rabbit conjugated to FITC (DakoCytomation) at 1∶400, goat anti-mouse Alexa Fluor 488 (Invitrogen) at 1∶1000, goat anti-rabbit conjugated to Cy3 (Jackson ImmunoResearch) at 1∶1000, donkey anti-guinea pig conjugated to TRITC (Jackson ImmunoResearch) at 1∶400, goat anti-human conjugated to Cy5 (GE Healthcare) at 1∶1000, goat anti-human conjugated to Cy3 (GE Healthcare) at 1∶1000. Slides were mounted in Prolong Gold (Molecular Probes). Slides were viewed using a Leica DMRA2 microscope and a 100× objective with epifluorescence, captured by a Hamamatsu digital CCD camera C4742-95 and Openlab 3.1.4 software and processed by Openlab 3.1.4, Volocity 5.5.1 and Adobe Photoshop 9.0.

### Preparation of the MI oocytes

Ovaries from wild-type, *Tex12^−/−^Sycp3^−/−^* and *Sycp1^−/−^Sycp3^−/−^* 4-weeks old mice were dissected, oocytes at the germinal vesicle stage isolated and fixed in methanol-acetic acid 3∶1 solution, as described previously [58]. To obtain oocytes at the metaphase I stage, cells were cultured for 6 hours. After fixation, oocytes were stained with DAPI and imaged using a Leica DMRA2 microscope, as described above.

### Staging of the oocytes

To determine the developmental stages of oocytes derived from *Sycp1^−/−^*, *Tex12^−/−^*, *Tex12^−/−^Sycp3^−/−^* and *Sycp1^−/−^Sycp3^−/−^* animals, we took advantage of the synchronous development that oocytes undergo in embryonic ovaries [59]. We labeled the axes and centromeres of chromosomes in oocytes derived from ovaries taken from animals at E16.5, E17.5, E18.5 and E19.5. The major fraction of oocytes found in E16.5 ovaries was classified as “early zygotene”, at E17.5 as “early pachyten”, at E18.5 as “late pachytene” and at E19.5 as “diplotene”. Briefly, zygotene oocytes displayed 40 distinct centromeres in a few groups and extended axial structures in close association. Early pachytene oocytes displayed joint axial structures and centromeres clustered in a few regions of the nucleus. In late pachytene oocytes, centromeres were evenly distributed in the nucleus and the chromosome axes were apparent but no longer aligned. In diplotene oocytes, the centromeres were clustered, but the chromosome axes had disintegrated.

**Quantifications** were performed using a Measurements module of the Volocity 5.5.1 software (Improvision) and ImageJ 1.43u software. Oocytes derived from mutant animals and their wild-type littermates were spread as described above, stained with different antibodies of interest plus STAG3 antibody to assess axis morphology and counterstained with DAPI. We used oocytes derived from E17.5 ovaries, as stages from zygotene to diplotene could be identified in the same sample. All slides stained with the same antibodies were processed simultaneously to minimize variation; images were taken with the same exposure times. Only oocytes with intact morphology (as judged by DAPI staining) and adequate spreading (nucleus diameter between 30 and 50 µm) were processed. The measurements were taken from one image, representing the focal plane for the whole cell. To quantify the intensity of γH2AX in the nucleus, the meiotic nuclei were outlined and the mean intensity of the γH2AX staining was measured by the Volocity 5.5.1 measurement module after background subtraction. We assumed that the protein concentration is directly proportional to the observed intensity of the immunofluorescent signal. To calculate the number of RAD51, DMC1, RPA, MSH4 foci, the chromosomal axes were outlined and the number of foci co-localizing with the axes was automatically counted by the Volocity 5.5.1 measurement module. To determine the strength of interference between MSH4 foci in early pachytene oocytes, the axial length between MSH4 foci was measured only in regions where the axes could be followed. The interfocal distances were analyzed by the Statistica 7.0 software, in order to obtain the maximum likelihood estimation of the shape parameter (ν of the gamma distribution. The number of cell used for the statistical analysis is shown in Table 3.

**Table 3:** The number of cells used for the statistical analysis of the recombination process.

	Wild-type	*Sycp1^−/−^*	*Tex12^−/−^*	*Sycp1^−/−^Sycp3^−/−^*	*Tex12^−/−^Sycp3^−/−^*
γH2AXmean intensity*	4/6/6/4	4/5/5/5	5/5/5/4	4/5/5/4	5/6/5/6
RAD51 foci number*	5/15/22/8	9/13/16/6	6/4/5/4	10/11/14/7	4/5/5/4
DMC1 foci number*	4/6/5/5/	4/5/9/5	4/6/5/4	4/5/7/6	4/4/5/4
RPA foci number*	4/5/5/4/	4/5/7/5	4/6/5/4	4/5/9/6	4/5/5/4
MSH4 foci number*	5/18/25/8	9/17/13/6	6/6/5/4	6/13/7/7	4/5/5/5
MSH4 foci interference**	4 (68)	6 (96)	-	7 (93)	-

*number of analyzed cells at the zygotene/early pachytene/late pachytene/diplotene stages.

**total number of analyzed axial intervals is in parentheses.

### Ovary sections

We collected ovaries from *Sycp1^−/−^* and *Sycp1^−/−^Sycp3^−/−^* animals at E16.5, when a majority of the oocytes in wild-type ovaries had reached the zygotene stage; at day 1 after birth (1dpp), when a majority of oocytes have entered the diplotene/dictyate transition; at 8 days after birth (8dpp), when a majority of oocytes have reached the dictyate stage; at 4 weeks after birth, when all follicle types have been formed; and finally at 8 weeks, when the ovaries have reached maturity. Ovaries were fixed in 4% formaldehyde for 4 hours, paraffin-embedded and sectioned at 5 µm. To count oocyte numbers in the ovary, each 5^th^ section from E16.5, 1dpp and 8dpp ovaries was immunostained for GCNA, and from 4-week- and 8-week-old animals, the sections were stained with eosin and hematoxylin, as described before [45]. The images were collected on Leica DMRA2 microscope. From 3 to 6 animals were analyzed for each genotype and each time point.

**Statistical analysis** was performed using Excel 2011 and Statistica 7.0.

## References

[1] Zickler D, Kleckner N (1999). Meiotic chromosomes: integrating structure and function.. Annu Rev Genet.

[2] Page SL, Hawley RS (2004). The genetics and molecular biology of the synaptonemal complex.. Annu Rev Cell Dev Biol.

[3] Yang F, Wang PJ (2009). The Mammalian synaptonemal complex: a scaffold and beyond.. Genome Dyn.

[4] Handel MA, Schimenti JC (2010). Genetics of mammalian meiosis: regulation, dynamics and impact on fertility.. Nat Rev Genet.

[5] de Vries FA, de Boer E, van den Bosch M, Baarends WM, Ooms M (2005). Mouse *Sycp1* functions in synaptonemal complex assembly, meiotic recombination, and XY body formation.. Genes Dev.

[6] Bolcun-Filas E, Costa Y, Speed R, Taggart M, Benavente R (2007). SYCE2 is required for synaptonemal complex assembly, double strand break repair, and homologous recombination.. J Cell Biol.

[7] Hamer G, Novak I, Kouznetsova A, Hoog C (2008). Disruption of pairing and synapsis of chromosomes causes stage-specific apoptosis of male meiotic cells.. Theriogenology.

[8] Bolcun-Filas E, Hall E, Speed R, Taggart M, Grey C (2009). Mutation of the mouse *Syce1* gene disrupts synapsis and suggests a link between synaptonemal complex structural components and DNA repair.. PLoS Genet.

[9] Schramm S, Fraune J, Naumann R, Hernandez-Hernandez A, Hoog C (2011). A novel mouse synaptonemal complex protein is essential for loading of central element proteins, recombination, and fertility.. PLoS Genet.

[10] Revenkova E, Jessberger R (2006). Shaping meiotic prophase chromosomes: cohesins and synaptonemal complex proteins.. Chromosoma.

[11] Nasmyth K, Haering CH (2009). Cohesin: its roles and mechanisms.. Annu Rev Genet.

[12] Daniel K, Lange J, Hached K, Fu J, Anastassiadis K (2011). Meiotic homologue alignment and its quality surveillance are controlled by mouse HORMAD1.. Nat Cell Biol.

[13] Fukuda T, Daniel K, Wojtasz L, Toth A, Hoog C (2010). A novel mammalian HORMA domain-containing protein, HORMAD1, preferentially associates with unsynapsed meiotic chromosomes.. Exp Cell Res.

[14] Shin YH, Choi Y, Erdin SU, Yatsenko SA, Kloc M (2010). *Hormad1* mutation disrupts synaptonemal complex formation, recombination, and chromosome segregation in mammalian meiosis.. PLoS Genet.

[15] Pelttari J, Hoja MR, Yuan L, Liu JG, Brundell E (2001). A meiotic chromosomal core consisting of cohesin complex proteins recruits DNA recombination proteins and promotes synapsis in the absence of an axial element in mammalian meiotic cells.. Mol Cell Biol.

[16] Yang F, De La Fuente R, Leu NA, Baumann C, McLaughlin KJ (2006). Mouse SYCP2 is required for synaptonemal complex assembly and chromosomal synapsis during male meiosis.. J Cell Biol.

[17] Yuan L, Liu JG, Zhao J, Brundell E, Daneholt B (2000). The murine *SCP3* gene is required for synaptonemal complex assembly, chromosome synapsis, and male fertility.. Mol Cell.

[18] Smith AV, Roeder GS (1997). The yeast Red1 protein localizes to the cores of meiotic chromosomes.. J Cell Biol.

[19] Webber HA, Howard L, Bickel SE (2004). The cohesion protein ORD is required for homologue bias during meiotic recombination.. J Cell Biol.

[20] Wang K, Wang M, Tang D, Shen Y, Qin B (2011). PAIR3, an axis-associated protein, is essential for the recruitment of recombination elements onto meiotic chromosomes in rice.. Mol Biol Cell.

[21] Storlazzi A, Xu L, Schwacha A, Kleckner N (1996). Synaptonemal complex (SC) component Zip1 plays a role in meiotic recombination independent of SC polymerization along the chromosomes.. Proc Natl Acad Sci U S A.

[22] Page SL, Hawley RS (2001). *c(3)G* encodes a *Drosophila* synaptonemal complex protein.. Genes Dev.

[23] Schild-Prufert K, Saito TT, Smolikov S, Gu Y, Hincapie M (2011). Organization of the Synaptonemal Complex During Meiosis in *Caenorhabditis elegans*.. Genetics.

[24] Higgins JD, Sanchez-Moran E, Armstrong SJ, Jones GH, Franklin FC (2005). The *Arabidopsis* synaptonemal complex protein ZYP1 is required for chromosome synapsis and normal fidelity of crossing over.. Genes Dev.

[25] Osman K, Sanchez-Moran E, Higgins JD, Jones GH, Franklin FC (2006). Chromosome synapsis in *Arabidopsis*: analysis of the transverse filament protein ZYP1 reveals novel functions for the synaptonemal complex.. Chromosoma.

[26] Costa Y, Speed R, Ollinger R, Alsheimer M, Semple CA (2005). Two novel proteins recruited by synaptonemal complex protein 1 (SYCP1) are at the centre of meiosis.. J Cell Sci.

[27] Hamer G, Gell K, Kouznetsova A, Novak I, Benavente R (2006). Characterization of a novel meiosis-specific protein within the central element of the synaptonemal complex.. J Cell Sci.

[28] Page SL, Khetani RS, Lake CM, Nielsen RJ, Jeffress JK (2008). *corona* is required for higher-order assembly of transverse filaments into full-length synaptonemal complex in *Drosophila* oocytes.. PLoS Genet.

[29] Liebe B, Alsheimer M, Hoog C, Benavente R, Scherthan H (2004). Telomere attachment, meiotic chromosome condensation, pairing, and bouquet stage duration are modified in spermatocytes lacking axial elements.. Mol Biol Cell.

[30] Kouznetsova A, Novak I, Jessberger R, Hoog C (2005). SYCP2 and SYCP3 are required for cohesin core integrity at diplotene but not for centromere cohesion at the first meiotic division.. J Cell Sci.

[31] Chicheportiche A, Bernardino-Sgherri J, de Massy B, Dutrillaux B (2007). Characterization of *Spo11*-dependent and independent phospho-H2AX foci during meiotic prophase I in the male mouse.. J Cell Sci.

[32] Bannister LA, Schimenti JC (2004). Homologous recombinational repair proteins in mouse meiosis.. Cytogenet Genome Res.

[33] Snowden T, Acharya S, Butz C, Berardini M, Fishel R (2004). hMSH4-hMSH5 recognizes Holliday Junctions and forms a meiosis-specific sliding clamp that embraces homologous chromosomes.. Mol Cell.

[34] Kolas NK, Cohen PE (2004). Novel and diverse functions of the DNA mismatch repair family in mammalian meiosis and recombination.. Cytogenet Genome Res.

[35] de Boer E, Heyting C (2006). The diverse roles of transverse filaments of synaptonemal complexes in meiosis.. Chromosoma.

[36] de Boer E, Dietrich AJ, Hoog C, Stam P, Heyting C (2007). Meiotic interference among MLH1 foci requires neither an intact axial element structure nor full synapsis.. J Cell Sci.

[37] Bishop DK, Zickler D (2004). Early decision; meiotic crossover interference prior to stable strand exchange and synapsis.. Cell.

[38] Khetani RS, Bickel SE (2007). Regulation of meiotic cohesion and chromosome core morphogenesis during pachytene in *Drosophila* oocytes.. J Cell Sci.

[39] Schwacha A, Kleckner N (1997). Interhomolog bias during meiotic recombination: meiotic functions promote a highly differentiated interhomolog-only pathway.. Cell.

[40] Woods LM, Hodges CA, Baart E, Baker SM, Liskay M (1999). Chromosomal influence on meiotic spindle assembly: abnormal meiosis I in female *Mlh1* mutant mice.. J Cell Biol.

[41] Lipkin SM, Moens PB, Wang V, Lenzi M, Shanmugarajah D (2002). Meiotic arrest and aneuploidy in MLH3-deficient mice.. Nat Genet.

[42] Guillon H, Baudat F, Grey C, Liskay RM, de Massy B (2005). Crossover and noncrossover pathways in mouse meiosis.. Mol Cell.

[43] Niu H, Wan L, Baumgartner B, Schaefer D, Loidl J (2005). Partner choice during meiosis is regulated by Hop1-promoted dimerization of Mek1.. Mol Biol Cell.

[44] Li XC, Bolcun-Filas E, Schimenti JC (2011). Genetic Evidence that Synaptonemal Complex Axial Elements Govern Recombination Pathway Choice in Mice.. Genetics.

[45] Wang H, Hoog C (2006). Structural damage to meiotic chromosomes impairs DNA recombination and checkpoint control in mammalian oocytes.. J Cell Biol.

[46] Woltering D, Baumgartner B, Bagchi S, Larkin B, Loidl J (2000). Meiotic segregation, synapsis, and recombination checkpoint functions require physical interaction between the chromosomal proteins Red1p and Hop1p.. Mol Cell Biol.

[47] Xu L, Weiner BM, Kleckner N (1997). Meiotic cells monitor the status of the interhomolog recombination complex.. Genes Dev.

[48] Bhalla N, Dernburg AF (2005). A conserved checkpoint monitors meiotic chromosome synapsis in *Caenorhabditis elegans*.. Science.

[49] Colaiacovo MP, MacQueen AJ, Martinez-Perez E, McDonald K, Adamo A (2003). Synaptonemal complex assembly in *C. elegans* is dispensable for loading strand-exchange proteins but critical for proper completion of recombination.. Dev Cell.

[50] MacQueen AJ, Colaiacovo MP, McDonald K, Villeneuve AM (2002). Synapsis-dependent and -independent mechanisms stabilize homolog pairing during meiotic prophase in *C. elegans*.. Genes Dev.

[51] Manheim EA, McKim KS (2003). The Synaptonemal complex component C(2)M regulates meiotic crossing over in *Drosophila*.. Curr Biol.

[52] Mehrotra S, McKim KS (2006). Temporal analysis of meiotic DNA double-strand break formation and repair in *Drosophila* females.. PLoS Genet.

[53] Ramesh MA, Malik SB, Logsdon JM (2005). A phylogenomic inventory of meiotic genes; evidence for sex in *Giardia* and an early eukaryotic origin of meiosis.. Curr Biol.

[54] Wilkins AS, Holliday R (2009). The evolution of meiosis from mitosis.. Genetics.

[55] Loidl J (2006). *S. pombe* linear elements: the modest cousins of synaptonemal complexes.. Chromosoma.

[56] Loidl J, Scherthan H (2004). Organization and pairing of meiotic chromosomes in the ciliate *Tetrahymena thermophila*.. J Cell Sci.

[57] Peters AH, Plug AW, van Vugt MJ, de Boer P (1997). A drying-down technique for the spreading of mammalian meiocytes from the male and female germline.. Chromosome Res.

[58] Kouznetsova A, Lister L, Nordenskjold M, Herbert M, Hoog C (2007). Bi-orientation of achiasmatic chromosomes in meiosis I oocytes contributes to aneuploidy in mice.. Nat Genet.

[59] Dietrich AJ, Mulder RJ (1983). A light- and electron microscopic analysis of meiotic prophase in female mice.. Chromosoma.

